# GIS-based assessment of spatial and temporal disparities of urban health index in Shenzhen, China

**DOI:** 10.3389/fpubh.2024.1429143

**Published:** 2024-09-03

**Authors:** Duan Yongheng, Xie Shan, Liu Fei, Tang Jinglin, Gong Liyue, Liu Xiaoying, Wen Tingxiao, Wang Hongrui

**Affiliations:** ^1^Shenzhen Health Development Research and Data Management Center, Shenzhen, Guangdong, China; ^2^National Defense Technology Strategic Research Think Tank of National University of Defense Technology, Changsha, Hunan, China; ^3^School of Life Sciences, Central South University, Changsha, China; ^4^Library of Central South University, Changsha, China

**Keywords:** urban health index, health inequalities, spatial autocorrelation analysis, highincome city, geographic in information system (GIS), Moran’s *I*

## Abstract

**Purpose:**

To explore the inter-regional health index at the city level to contribute to the reduction of health inequalities.

**Methods:**

Employed the health determinant model to select indicators for the urban health index of Shenzhen City. Utilized principal component analysis, the weights of these indicators are determined to construct the said health index. Subsequently, the global Moran’s index and local Moran’s index are utilized to investigate the geographical spatial distribution of the urban health index across various administrative districts within Shenzhen.

**Results:**

The level of urban health index in Shenzhen exhibits spatial clustering and demonstrates a positive spatial correlation (2017, Moran’s *I* = 0.237; 2019, Moran’s *I* = 0.226; 2021, Moran’s *I* = 0.217). However, it is noted that this clustering displays a relatively low probability (90% confidence interval). Over the period from 2017 to 2019, this spatial clustering gradually diminishes, suggesting a narrowing of health inequality within economically developed urban areas.

**Conclusion:**

Our study reveals the urban health index in a relatively high-income (Shenzhen) in a developing country. Certain spatially correlated areas in Shenzhen present opportunities for the government to address health disparities through regional connectivity.

## 1 Introduction

Health serves as the foundation for human survival and development, impacting not only the quality of life of individuals but also national security and societal stability. Health inequality can be defined as variations in health status across individuals within a population. It may have a potential relationship with various socioeconomic outcomes (1). Some health inequalities are due to differences in natural resources and geography. However, some health inequalities are due to social injustices, such as discriminatory practices based on race, gender and culture and the failure of societies to provide basic health-care services to all, which are considered health inequities. Health inequality connotes a larger scope than health inequity, and health equity is the foundation of what should be done to promote health equality (2). Despite improvements in national health levels due to advancements in socio-economic factors and medical technology, disparities in health benefits persist, leading to intra-national and international health inequalities (3, 4). Presently, health inequality has emerged as a core issue affecting human development (5–7). The World Health Organization (WHO) strongly advocates for narrowing the health disparities among different population groups and regions within countries, with many nations prioritizing health inequality as a key societal concern (5).

The assessment of inequality has become an urgent matter, with the Urban Health Index (UHI) providing a metric for evaluating the equality of health opportunities. While numerical indicators alone may not fully capture the nuances of population health, they offer a comparative approach and provide clear evidence of disparities and inequalities. Various methodologies exist internationally for calculating Urban Health Indices, with the majority relying on the Delphi method to determine indicator weights through expert consensus (8, 9). Although numerous health index systems exist (10, 11), overarching systems may have limited applicability to different countries and cities (12). Unlike previous studies that directly obtained health index values from specific databases (13, 14), this paper constructs the Urban Health Index of Shenzhen based on existing research and the Health Determinants Model (HDM), objectively reflecting the equality of health opportunities for its residents. The HDM can be effectively categorized into four principal groups: social determinants, healthcare system characteristics, disease-inducing behaviors, and health outcomes, which provided a comprehensive approach for epidemiologists, public health professionals, and policymakers to analyze and understand the complex interplay between these factors (15). The health index in this paper is based on socio-economic and environmental factors and aims to find ways to reduce health inequalities by exploring the linkages of the health index between regions. This approach helps avoid data deficiencies and enhances specificity and accuracy.

As one of China’s most developed cities, Shenzhen is designated as a Special Economic Zone, a national economic center, and a national innovation-oriented city by the State Council (16), experiencing rapid development. It is a representative achievement of China’s booming economy since the reform and opening up and is a fast-growing new city. The development paths and patterns of new cities like Shenzhen are better adapted to the contemporary economic and technological environment. Its approach to city building and health equity promotion strategies are especially valuable for developing cities to learn from. Therefore, investigating the disparities and changes in its Urban Health Index is highly representative. Previous studies mainly explored health inequality within China (17, 18) or analyzed differences between rural and urban areas (19, 20), with limited research on the health equity of various administrative regions within a developed city. This paper explores the spatial correlation of the Shenzhen Urban Health Index to gain a better understanding of regional health disparities, providing valuable insights for reducing health disparities among different administrative regions within the city. Thus, this manuscript selected indicators based on the HDM, employs principal component analysis to determine weights, constructs the Urban Health Index of Shenzhen, visualizes it using Geographic Information System (GIS) technology, and analyzes differences and changes among different regions, aiming to provide guidance for reducing health inequality in the urban development process.

## 2 Materials and methods

### 2.1 Measurement of urban health index

The Health Determinants Model (21), outlines a conceptual framework for understanding the main determinants of health. It categorizes various health determinants into four distinct categories related to ‘policy level’ in public health, including individual lifestyle factors, social networks, living and working conditions, and socio-economic, cultural, and environmental conditions. This model emphasizes the consideration of different intervention levels in health policy-making, aiming to enable public health institutions to address all relevant policy aspects when dealing with specific health issues. Not only does this model simplify the conceptualization of health determinants, but it also provides theoretical guidance for promoting proactive health and formulating disease control interventions. With advancements in science, technology, and biomedical sciences, understanding of the HDM continues to evolve. For instance, modern interpretations of the model emphasize the role of the natural environment as the ecological foundation supporting human life and health, where the biosphere, landscape, and natural environment serve as the basis for health and well-being (22).

Therefore, this paper constructs the Urban Health Index of Shenzhen based on the HDM, combined with statistical data from various administrative districts of Shenzhen. It selects nine indicators from the years 2017, 2019, and 2021, including *per capita* public green space area, average regional environmental noise level, Goodness rate of Air Quality Index (AQI), *per capita* disposable income, *per capita* GDP, health expenditure, number of health institutions, number of health technical personnel, and number of medical insurance participants. These indicators reflect the local living environment, medical conditions, and economic development, which influence residents’ equal access to health opportunities. These indicators have been previously demonstrated as determinants of health levels (23–27).

### 2.2 Method

#### 2.2.1 Principal component analysis

To standardize the indicators of the UHI of Shenzhen, which have different units, the range standardization method was employed. All indicators were normalized to range from 0 (worst) to 1 (best) using Equation 1. For ease of expression in subsequent analysis, symbols were used to represent each indicator. To avoid the correlation of the original data and overemphasis on certain factors, and to more objectively reflect the data representing the population’s health status, we chose PCA to construct the UHI. The standardized data of the nine indicators were then imported into SPSS 26.0 software for Principal Component Analysis (PCA). Data sources include the Statistical Yearbooks and Statistical Bulletins of various districts in Shenzhen. The data is compiled by the Shenzhen Municipality Bureau of Statistics and Survey Office of the National Bureau of Statistics in Shenzhen, which comprehensively and systematically introduces the situation of the national economy and social development of Shenzhen, with the main indicators highlighting the achievements of different aspects of the economy and society in different years. The main indicators highlight the achievements of Shenzhen’s economy and society in different years.

The Kaiser-Meyer-Olkin (KMO) measure was used as a diagnostic tool to assess the suitability of the selected indicator data for principal component analysis. The KMO value ranges from 0 to 1, with values closer to 1 indicating that the selected indicator data are more suitable for PCA, resulting in better analysis outcomes. Principal components that can summarize the overall data were extracted. The component matrix and initial eigenvalues represent the weights of different indicator data affecting health status, from which equations for each principal component can be derived. Subsequently, the weight coefficients were calculated based on the proportions of the eigenvalues of two principal components to the sum of the eigenvalues of all principal components. This facilitated the computation of the UHI for the ten administrative districts of Shenzhen, which served as spatial analysis data.


(1) $$Normalization=\frac{\begin{array}{l} \mathrm{Empirical values}-\\ \mathrm{minimum theoretical value} \end{array}}{\begin{array}{l} \mathrm{maximum theoretical value}-\\ \mathrm{minimum theoretical value}\; \end{array}}$$

#### 2.2.2 GIS technology

GIS technology is an essential tool for acquiring, processing, storing, analyzing, and applying geographic spatial data. It integrates map visualizations with geographical data analysis, providing users with data support for decision-making in an intuitive manner (28). With the rapid development of information technology, GIS technology continues to advance. It integrates with advanced technologies such as database technology, internet technology, and virtual reality technology, enabling its application in various public health sectors such as responding to public health emergencies, disease surveillance, and prevention (29–31).

#### 2.2.3 Spatial autocorrelation analysis

Spatial autocorrelation analysis primarily examines whether the attribute values of a particular feature are significantly correlated with those of neighboring features in the spatial domain, aiming to measure the correlation and heterogeneity of the same attribute across different spatial units. It provides an effective method for analyzing the spatial distribution patterns and variations in spatial disparities of attribute values across different regions. Spatial autocorrelation analysis is divided into global spatial autocorrelation and local spatial autocorrelation. The former is utilized to describe the spatial characteristics of attribute values across the entire study area, with the Global Moran’s I value revealing whether the distribution of resident health conditions exhibits clustering. The latter is employed to determine the spatial association and variation of the same attribute between each spatial location and its adjacent neighbors within the study area. In this study, Moran scatterplots are employed to represent the degree of spatial correlation. Moran scatterplots are used to examine the differences between objects in the study area, and their advantage lies in their ability to clearly differentiate study objects from adjacent spatial units. Moran scatterplots are expressed using Cartesian coordinates, with the horizontal axis representing standardized observed values and the vertical axis representing standardized local spatial autocorrelation analysis Moran’s I values. There are four quadrants: ‘High-High (HH),’ ‘High-Low (HL),’ ‘Low-Low (LL),’ and ‘Low-High (LH).’

Moran’s *I* index is an important indicator used to explore the potential interdependence of observed data within the same distribution area (32), employed to investigate whether there is spatial correlation in the UHI of Shenzhen. change to: It is mainly divided into global Moran’s *I* and local Moran’s *I*, with the calculation formulas as (Equations 2 and 3) perspectively.


(2) $$\mathrm{Global}\;\mathrm{Moran}’s\;I=\frac{n\overset{n}{\underset{i=1}{\sum }}\overset{n}{\underset{j=1}{\sum }}W_{ij}\left(X_{i}-\bar{X}\right)\left(X_{j}-\bar{X}\right)}{\overset{n}{\underset{i=1}{\sum }}\overset{n}{\underset{j=1}{\sum }}W_{ij}\overset{n}{\underset{i=1}{\sum }}{\left(X_{i}-\bar{X}\right)}^{2}},$$

(3) $$\mathrm{Local}\;\mathrm{Moran}’s\;I=\frac{n\left(X_{i}-\bar{X}\right)}{\overset{n}{\underset{i=1}{\sum }}\left(X_{i}-\bar{X}\right)}\overset{n}{\underset{j=1,j\neq i}{\sum }}W_{ij}\left(X_{j}-\bar{X}\right),$$

Note: In the equation, ‘n’ represents the number of spatial units contained within the study area, denotes the average value of the attribute represents the spatial statistics between spatial locations i and j, and W represents the adjacency relationship between spatial locations. The range of Moran’s *I* values is [−1, 1]. At a given level of significance testing, a positive Moran’s *I* value indicates spatial clustering of health levels; conversely, a negative Moran’s *I* value suggests significant differences in health levels among neighboring areas, with regions exhibiting either lower or higher health levels demonstrating significant spatial dispersion.

## 3 Results

### 3.1 Urban health index of Shenzhen

Using SPSS 26.0 software, the KMO measure was employed to validate all indicator data. The KMO value for the indicator data was found to be 0.663, which exceeds 0.5, indicating that the selected indicator data are suitable for PCA. According to Results of the PCA, both Component 1 and Component 2 have eigenvalues greater than 1, with contribution rates reaching 47.637 and 23.448%, respectively. Therefore, the extracted principal components F1 and F2 represent a total of 71.085% of all information, indicating that the extracted principal components are representative. The component matrix of the indicator data extracted by principal component analysis is shown in Table 1. Based on statistical analysis methods, change to: the (Equations 4 and 5) for the two principal components can be derived as follows:


(4) $$\begin{array}{l} \mathrm{F1}=0.944^{*}X_{1}+0.933^{*}X_{2}+0.919^{*}X_{3}+0.908^{*}X_{4}\\ +{\left(-0.662\right)}^{*}X_{5}+0.015^{*}X_{6}+0.559^{*}X_{7}\\ +{\left(-0.035\right)}^{*}X_{8}+{\left(-0.309\right)}^{*}X_{9} \end{array}$$

(5) $$\begin{array}{l} \mathrm{F2}=0.024^{*}X_{1}+0.08^{*}X_{2}+{\left(-0.003\right)}^{*}X_{3}+{\left(-0.122\right)}^{*}X_{4}\\ +0.107^{*}X_{5}+0.838^{*}X_{6}+0.732^{*}X_{7}+0.71^{*}X_{8}\\ +0.586^{*}X_{9} \end{array}$$

**Table 1:** Component matrix of indicator data.

Indicators	Representative symbol	Component 1	Component 2
Number of participants in medical insurance	X1	0.944	0.024
Number of health technicians	X2	0.933	0.08
Expenditure on health	X3	0.919	−0.003
Number of health institutions	X4	0.908	−0.122
Public green space *per capita*	X5	−0.662	0.107
GDP *per capita*	X6	0.015	0.838
*Per capita* disposable income	X7	0.559	0.732
Average regional ambient noise	X8	−0.035	0.71
Goodness Rate of AQI	X9	−0.309	0.586

The equation for the overall resident health index is:


(6) $$F=F_{1}*0.67+F_{2}*0.33$$

The urban health index for the 10 administrative units in Shenzhen, as calculated (Equation 6), is presented in Table 2. This index will be utilized as the data for conducting spatial autocorrelation analysis.

**Table 2:** UHI of Shenzhen in 2017, 2019, and 2021.

Districts	Health index (2017)	Health index (2019)	Health index (2021)
Futian District	1.965	2.376	2.874
Luohu District	1.166	1.398	1.711
Nanshan District	1.748	2.212	2.421
Yantian District	0.200	0.483	0.550
Baoan District	1.481	2.105	2.664
Longgang District	1.719	2.117	2.244
Guangming District	0.322	0.459	0.415
Pingshan District	0.197	0.394	0.738
Longhua District	0.628	0.939	1.301
Dapeng New District	−0.119	−0.051	0.060

It is observed that the resident health index has gradually increased from 2017 to 2021. In 2021, Futian District had the highest resident health index at 2.874, while Dapeng New District had the lowest resident health index at 0.060, indicating a noticeable disparity.

### 3.2 Spatial distribution of UHI

**Figure 1:**
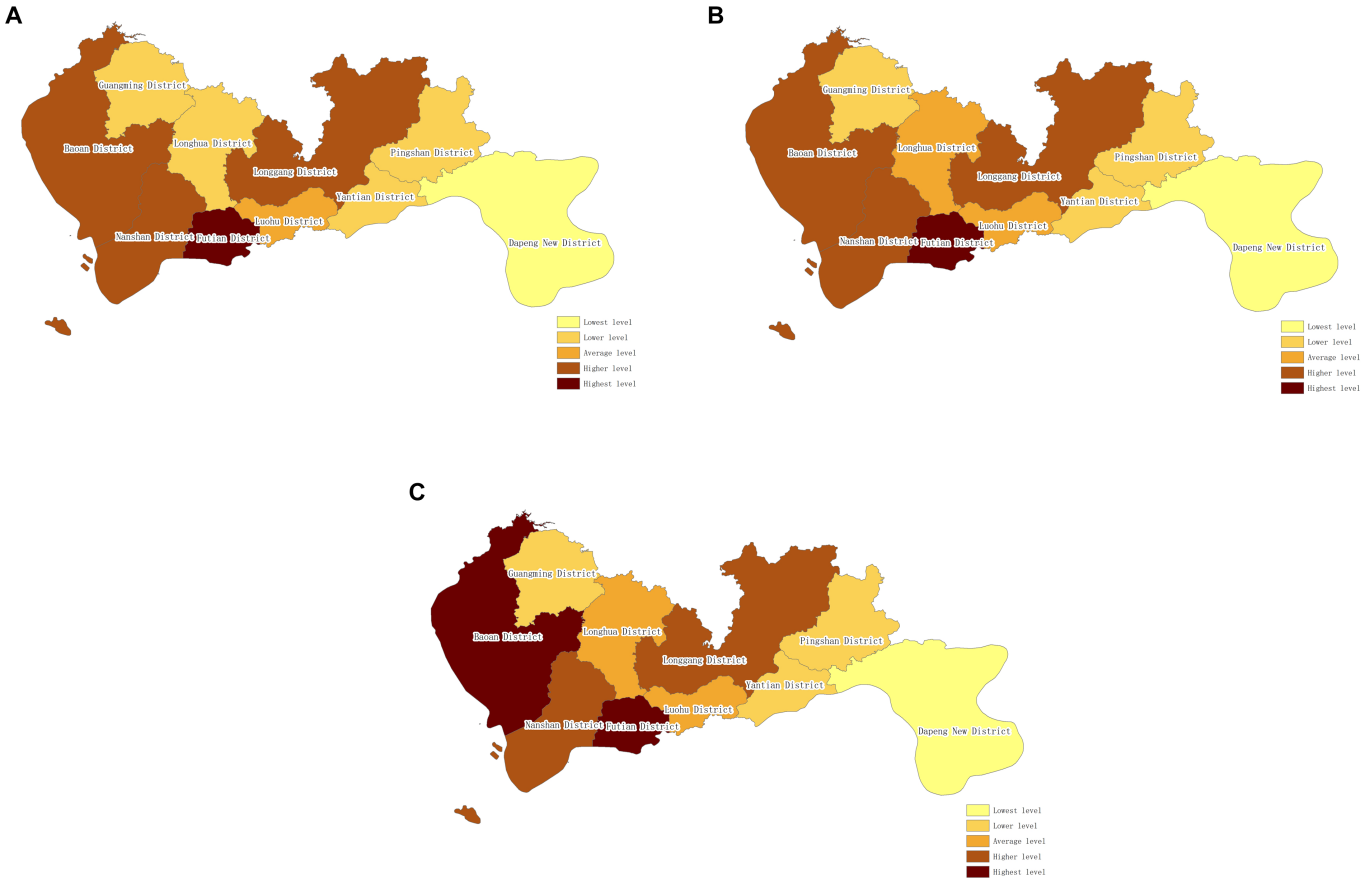
Spatial distribution of UHI of Shenzhen (from 2017 to 2021). **(A)** Spatial distribution of UHI of Shenzhen (2017). **(B)** Spatial Distribution of UHI of Shenzhen (2019). **(C)** Spatial distribution of UHI of Shenzhen (2021).

In order to directly illustrate the changing trends in the spatial distribution characteristics of resident health status in China, the method of equal intervals in ArcGIS 10.2 software was employed to classify provinces into different levels and display them in different colors. The 10 administrative regions were categorized into five types: high-level areas, relatively high-level areas, medium-level areas, relatively low-level areas, and low-level areas. The changes in resident health status in Shenzhen from 2017 to 2021 are shown in Figure 1.

From the figure, it can be observed that there were relatively minor changes in the resident health status in Shenzhen from 2017 to 2021. Provinces with high-level resident health status remained relatively stable, with increases in resident health index observed in Longgang District and Bao’an District. Longgang District transitioned from a relatively low-level area to a medium-level area, while Bao’an District changed from a relatively high-level area to a high-level area. In 2017, there were five areas with resident health status at medium level or above, accounting for 50%, which increased to 60% by 2021.

### 3.3 Disparities in UHI across regions

Utilizing the Resident Health Status Index of Shenzhen, a global spatial autocorrelation analysis was conducted using ArcGIS 10.2 software. The results of the global Moran’s I calculation are presented in Table 3. The Moran’s I values for the resident health index in 2017, 2019, and 2021 were all greater than 0, with normal Z-statistic values exceeding 1.65. This indicates a positive spatial correlation in the overall resident health status in China, implying that provinces with higher or lower resident health levels tend to cluster rather than exhibit random distribution. The *p*-values were all less than 0.1, indicating statistical significance at the 90% confidence interval.

**Table 3:** Global Moran’s *I* of UHI of Shenzhen.

Years	Moran’s *I*	*Z*-value	*P*-value
2017	0.237	1.692	0.052
2019	0.226	1.757	0.053
2021	0.217	1.804	0.058

The fluctuation in the global Moran’s *I* values indicates that from 2017 to 2021, the range of variation in the global Moran’s *I* values was 0.02. This suggests relatively minor changes in the spatial relationships of resident health status among provinces, indicating a tendency toward stability in resident health status spatial changes during this period. Additionally, the gradually decreasing values of the global Moran’s *I* over the 5 years imply a reduction in the clustering of the resident health index in Shenzhen, indicating a decreasing trend in its spatial autocorrelation over the study period.

**Figure 2:**
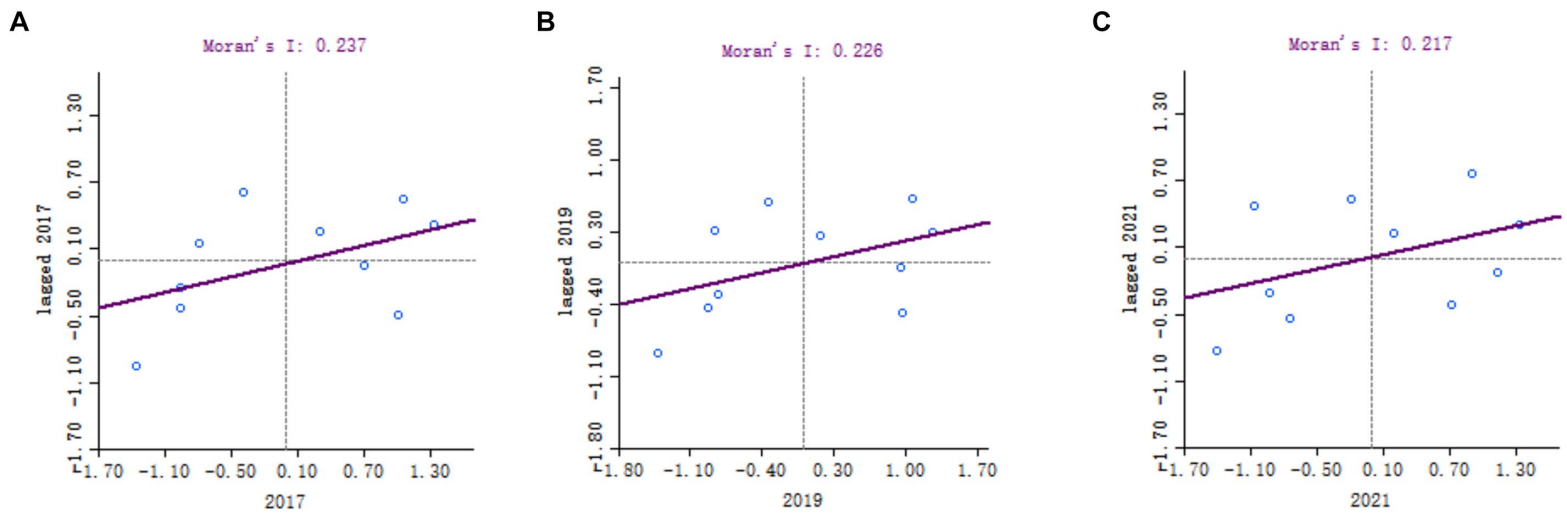
The Moran scatterplot of each administrative district in Shenzhen (from 2017 to 2021). The horizontal axis represents standardized values of observations, while the vertical axis represents standardized local spatial autocorrelation analysis Moran’s *I* values. The four quadrants denote ‘High-High (HH)’ for high values neighboring high values, ‘High-Low (HL)’ for high values neighboring low values, ‘Low-Low (LL)’ for low values neighboring low values, and ‘Low-High (LH)’ for low values neighboring high values.

To further understand the local conditions of resident health status in Shenzhen, local spatial autocorrelation analysis was conducted using spatial statistical analysis software (GeoDa). The Moran scatterplot of each administrative district in Shenzhen is depicted in Figure 2. In the plot, the majority of points fall within the first and third quadrants, indicating positive spatial autocorrelation among them. This implies that the resident health status in these districts tends to influence neighboring areas to a certain extent. Fewer administrative districts are located in the second and fourth quadrants, indicating negative spatial autocorrelation among them.

**Figure 3:**
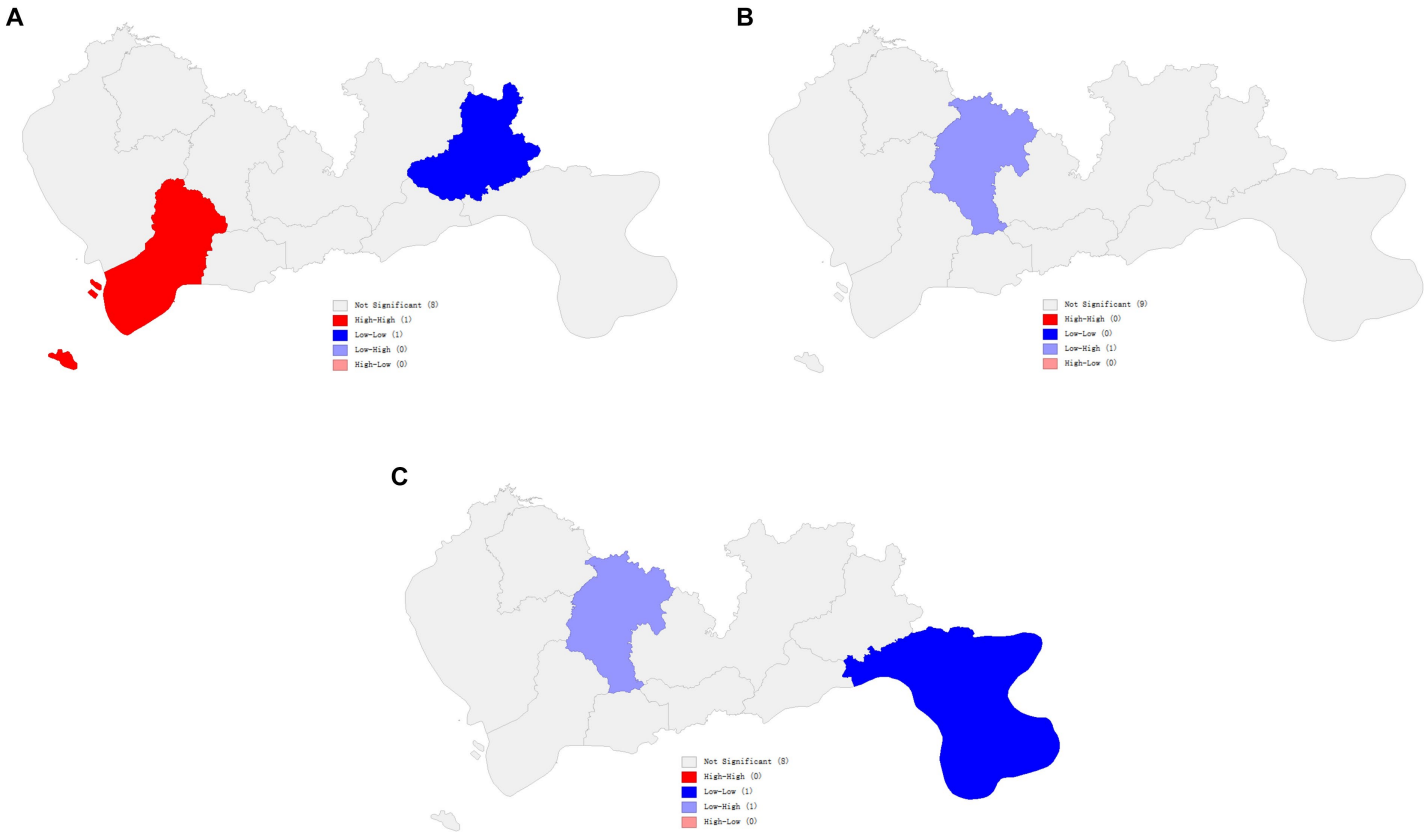
The LISA cluster map of Shenzhen (from 2017 to 2021). **(A)** The LISA cluster map of Shenzhen (2017). **(B)** The LISA cluster map of Shenzhen (2019). **(C)** The LISA cluster map of Shenzhen (2021).

To provide a more intuitive understanding of the clustering patterns of residents’ health conditions in Shenzhen over different years, LISA cluster maps are used to display the statistical results. The LISA cluster map, as shown in Figure 3, illustrates that in 2017, Nanshan District and Pingshan District exhibit statistically significant positive spatial autocorrelation. Their spatial autocorrelation types are High-High and Low-Low, respectively. This suggests that Nanshan District and its surrounding administrative areas have relatively higher levels of health index, while Pingshan District and its surrounding administrative areas have relatively lower levels of health index. In 2019, Longgang District shows significant negative spatial autocorrelation with its surrounding areas in terms of health index. In 2021, Dapeng New District exhibits clustering phenomenon with its surrounding administrative areas, with health index levels relatively low. Overall, the residents’ health index in Shenzhen demonstrates characteristics of local spatial correlation.

## 4 Discussion

This study utilized the Health Determinants Model to select indicators and employed the PCA to determine weights, constructing the UHI of Shenzhen. Subsequently, spatial autocorrelation analysis was conducted to explore spatial disparities and trends in residents’ health conditions. Overall, the UHI in different regions of Shenzhen exhibited clustering tendencies, indicating that health spatial inequality persists even in relatively developed cities in China. Research by Jinqi Jiang et al. has found that there exists health inequality in rural areas in China, favoring the affluent (33). Other studies have also demonstrated significant health disparities between rich and poor populations across China (34, 35). Our findings reveal that while health spatial inequality exists in developed cities, its likelihood, as indicated by our *p*-values falling within the 90% confidence interval, is relatively lower compared to other studies, suggesting reduced health inequality in economically developed regions.

Moreover, our study found a gradual decline in the global Moran’s *I* from 2017 to 2021 (from 0.237 to 0.217), indicating a diminishing clustering trend and a reduction in health inequality in Shenzhen. This differs from the results of other studies which indicated an expansion followed by contraction of health inequality indices (36–38). Possible reasons for this discrepancy include: (1) our study focusing on Shenzhen rather than China as a whole, and (2) differences in the time frames studied, with our analysis covering the period from 2017 to 2021. Currently, the Chinese government is implementing a series of measures to significantly reduce health inequality (39). The results of our study may benefit from these policies initiated by the Chinese government in October 2016.

In terms of local spatial relationships, most areas in Shenzhen demonstrated positive spatial autocorrelation in residents’ health index, indicating a radiation effect across different regions. This finding aligns with previous literature (40, 41). It is noteworthy that Longgang District exhibited a ‘Low-High’ spatial autocorrelation type in 2019 and 2021. Considering the earlier findings of changes in health index levels, with Longgang District transitioning from a lower to a moderate level in 2019, this result may be attributed to positive influences from neighboring areas.

To enhance the connectivity between high-UHI and low-UHI districts in Shenzhen, several strategies can be employed. Firstly, cooperation and resource exchange between different administrative regions should be strengthened. Secondly, a series of targeted support policies can be developed, high-UHI districts are recommended to assist those with lower health indices through medical resource support, health governance strategies, and implementable health promotion methods. Lastly, addressing fiscal imbalances between regions through increased transfer payments can focus on supporting areas with lower health indices.

This study has several limitations: (1) It relied solely on objective government statistical data to reflect the health index levels of administrative districts, lacking residents’ self-perceived health status. Future research could incorporate survey data to obtain more comprehensive insights into residents’ health inequality. (2) Only the overall UHI spatial relationships were investigated, without examining each indicator separately. Future studies could explore individual indicators to better understand the nuances of health index inequalities among urban residents. (3) To more objectively reflect the impact of the social environment on residents’ health, we primarily constructed the health index from the perspectives of socioeconomic factors, environmental factors, and healthcare resources. This approach may overlook some other factors, such as residents’ emotions and social relationships. Future research could incorporate residents’ self-reported health indices along with the data-driven socioeconomic indices to further refine the index construction.

## 5 Conclusion

This study utilized the Health Determinants Model and previous research to select indicators for the UHI of Shenzhen. The PCA was employed to determine weights, constructing the UHI. Spatial correlation analysis was then conducted to explore the geographical distribution of health indices among different administrative districts. The study found that the level of the UHI in Shenzhen exhibited spatial clustering and positive spatial correlation, although this clustering tendency was relatively low. Furthermore, this spatial clustering decreased gradually from 2017 to 2019.

Based on these conclusions, relevant government departments could establish a universal health index to analyze the uneven distribution of health across different regions. This would facilitate targeted measures to address health inequalities, leveraging positive spatial correlations to improve the situation. Additionally, enhancing regional collaboration between areas with high and low health index levels could benefit regions with lower health index levels.

## Data Availability

Publicly available datasets were analyzed in this study. This data can be found here: https://tjj.sz.gov.cn/.
